# Contributions of age-related alterations of the retinal pigment epithelium and of glia to the AMD-like pathology in OXYS rats

**DOI:** 10.1038/srep41533

**Published:** 2017-01-30

**Authors:** Darya V. Telegina, Oyuna S. Kozhevnikova, Sergey I. Bayborodin, Nataliya G. Kolosova

**Affiliations:** 1 Institute of Cytology and Genetics SB RAS, Novosibirsk 630090, Russia; 2 Novosibirsk State University, Novosibirsk 630090, Russia

## Abstract

Age-related macular degeneration (AMD) is a major cause of blindness in developed countries, and the molecular pathogenesis of early events of AMD is poorly understood. It is known that age-related alterations of retinal pigment epithelium (RPE) cells and of glial reactivity are early hallmarks of AMD. Here we evaluated contributions of the age-related alterations of the RPE and of glia to the development of AMD-like retinopathy in OXYS rats. We showed that destructive alterations in RPE cells are a primary change during the development of retinopathy in OXYS rats. Furthermore, a defect of retinal maturation and decreased immune function at the preclinical stage of retinopathy were observed in OXYS rats in addition to the impairment of RPE cell proliferation and of their capacity for division. At the active stage of the disease, the atrophic alterations increased, and reactive gliosis was observed when disease progressed, but immune function stayed weakened. Unexpectedly, we did not observe migration of microglia and macrophages into the photoreceptor layer. These results and the wide spectrum of age-related retinal alterations in humans as well as individual differences in the risk of AMD may be attributed to genetic factors and to differences in the underlying molecular events.

Age-related macular degeneration (AMD) is a complex neurodegenerative disease with both genetic and environmental risk factors and remains a major cause of irreversible blindness affecting elderly people worldwide. Despite considerable progress in understanding of the pathogenesis of AMD, there are some unexplored areas, especially the molecular pathogenesis of early events in AMD. Degeneration of retinal pigment epithelium (RPE) cells is one of the hallmarks of AMD pathogenesis^1^,^2^, and in most cases, appears to follow accumulation of lipofuscin and sub-RPE deposits, including drusen^3^. The RPE performs numerous functions necessary for the choroid and photoreceptors, including phagocytosis of outer photoreceptor segments, absorption of excess light, processing of retinoids for phototransduction (visual cycle), maintenance of the blood–retina barrier, and secretion of growth factors, cytokines, and lipoprotein particles.

There is growing evidence that retinal glia become activated in the course of degenerative retinal diseases, thus playing a pivotal role in the initiation and propagation of a neurodegenerative process^4^,^5^. The retina contains three major classes of glia – Müller cells, astrocytes and a resident subpopulation of microglial cells – which share many functions within the retina and play a role in the metabolism of neurons, which is affected by their distribution and physiological state. Numerous studies have shown reactive gliosis involving Müller cells in the retina of vertebrates in response to various retinal pathologies including AMD^6^,^7^. There are reports of upregulation of intermediate filaments like vimentin, glial fibrillary acidic protein (GFAP) and nestin in Müller cells and astrocytes during AMD^8^. In addition, Müller cells secrete various proteins that can impair the blood–retina barrier and can increase production of cytokines, chemokines, and the complement cascade in pathological conditions and thereby can contribute to this degeneration of retina^9^,^10^. The primary resident immune cells in the retina are microglia, which carry out constant and dynamic immune surveillance of the extracellular environment and execute adaptive-immunity functions under conditions of tissue injury. Microglia are normally absent from the outer retina, the site affected by AMD^11^, but migration and infiltration of microglial cells into the outer retina under conditions of advanced age and disease implicate microglia in the neuroinflammatory aetiology of AMD. Activated microglial cells have high capacity for phagocytosis and express a number of pro- and anti-inflammatory molecules^12^. Maladaptive inflammatory responses of microglia contribute to the progression of various chronic neurodegenerative diseases, promote cell death^13^ and can to lead to degeneration of photoreceptors^14^. Numerous clinical and basic studies have implicated age-related alterations of the RPE layer and glial dysfunction in the development AMD^15^, but the mechanisms of the transition of normal age-related changes to the pathological phenotypes in AMD are incompletely understood.

Because in humans, the research on the pathogenesis of AMD, especially its early stages, is problematic, there is a need for animal models that closely mirror the human eye pathology. There is evidence that a suitable experimental model of AMD is senescence-accelerated OXYS rats, which spontaneously develop a phenotype similar to human age-related disorders including AMD-like retinopathy^16^,^17^,^18^,^19^,^20^. Retinopathy that develops in OXYS rats already at a young age corresponds (in terms of clinical manifestations and morphological characteristics) to the dry atrophic form of AMD in humans^17^,^21^. Nonetheless, neovascularisation develops in some (~10–20%) of these rats with age. The clinical signs of AMD-like retinopathy appear by the age of 3 months in 100% of OXYS rats against the background of a reduction in the transverse area of the RPE and impairment of choroidal microcirculation^16^. Significant pathological changes in the RPE as well as clinical signs of advanced stages of retinopathy are evident in OXYS rats older than 12 months. These changes manifest themselves as excessive accumulation of lipofuscin and amyloid in the RPE regions and whirling extensions of the basement membrane into the cytoplasm. Just as the dry form of human AMD, the initial alterations in the RPE cells later lead to atrophy of the choriocapillaris and a complete loss of photoreceptor cells in the OXYS retina by age 24 months^17^,^19^,^22^,^23^. This animal model is successfully used to study the pathways and molecular alterations implicated in the development and progression of these disorders as well as to test new therapeutic interventions^21^,^22^,^23^.

In this study, we investigated contributions of the age-related alterations of the RPE and of glia to the development of AMD-like pathology in OXYS rats. We compared these rats with the control Wistar rats and OXYS rats at different stages of the disease, including pre-clinical stage.

## Results

### RPE atrophy is characteristic of OXYS rats

**Figure 1:**
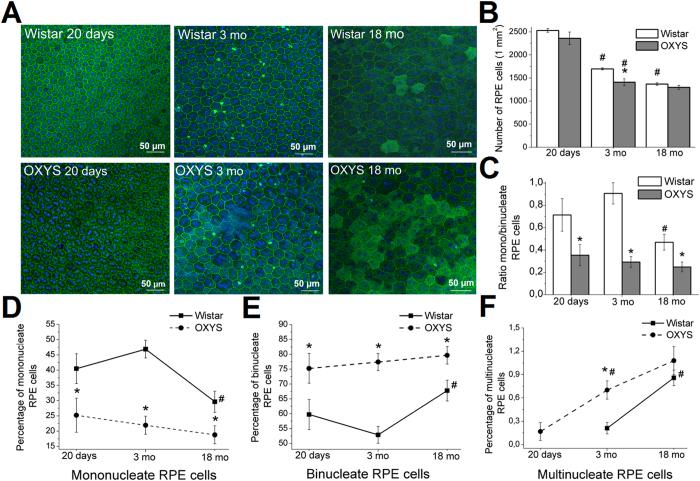
Analysis of alterations of RPE cells as a function of age in the retina of Wistar and OXYS rats. (**A**) Confocal images of a flat mount of Wistar (upper panel) and OXYS rat RPE (lower panel), showing the changes in RPE cells during normal aging (Wistar rats) and during the development of retinopathy (OXYS rats). Phalloidin was used for the RPE staining. Quantitative analysis of cell density of RPE (**B**), the ratio of the numbers of mono-/binucleate RPE cells (**C**), the percentages of mononucleate (**D**), binucleate (**E**), and multinucleate cells (**F**) per 1 mm^2^ in the central zone of the retina of OXYS and Wistar rats. The data are shown as mean ± SEM; *p < 0.05 for differences between the strains; ^#^p < 0.05 for age-related differences within a strain.

To evaluate age-associated changes in the morphology of RPE cells, especially during the development of AMD-like retinopathy, we examined the RPE cell monolayer in Wistar and OXYS rats a) at the age of 20 days when retinal maturation is complete and signs of the disease are absent in the retina of OXYS rats; b) at the age of 3 months, i.e. in the period of active manifestation of clinical signs of retinopathy and c) at age 18 months: during the active disease progression in OXYS rats. Using scanning confocal microscopy, we analysed only the central zone of the RPE, which is in close proximity to the exit site of the optic nerve (Fig. 1A).

With age, there was a gradual decrease in the density of RPE cells in both Wistar and OXYS rats. Only in OXYS rats did the density of RPE cells at age 3 months become 41% less than that in 20-day-old rats of this strain (p < 0.001), lower than in 3-month-old Wistar rats (p < 0.05) and similar to the RPE cell density in 18-month-old Wistar rats (Fig. 1B).

We observed atrophic changes of RPE cells in OXYS rats already at age 3 months and detected hypertrophy and hyperplasia along with the presence of auto-fluorescent content – lipofuscin granules – in some RPE cells. In addition, the nuclei of RPE cells in OXYS rats often appeared condensed and were located at the cell periphery. Some cells showed disturbances of the hexagonal structure (tetrahedral and polygonal cells), without disrupting an orderly mosaic; some authors regard these cells as healthy, normally functioning cells^24^ (Fig. 1A).

**Figure 2:**
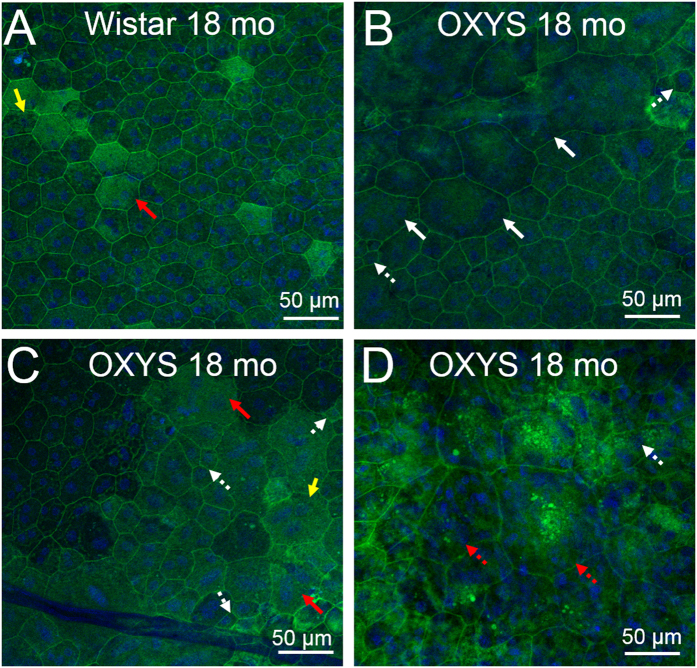
The destructive and atrophic alterations of RPE cells in Wistar and OXYS rats at the age of 18 months. Phalloidin was used for RPE staining. (**A**) Confocal images of a flat-mount of a Wistar rat RPE. The yellow arrows indicate polyploid cells. (**B**) The hypertrophy (white arrows) and hyperplasia (white dotted arrows) in OXYS rats during active progression of the disease. (**С**) Accumulation of lipofuscin granules in the disintegrating RPE cells in OXYS rats (red arrows). (**D**) Changes of the cell shape (red dotted arrow) disrupt an orderly mosaic in OXYS rats at age 18 months.

In the period of active disease progression, all the above-mentioned alterations increased, and some cells completely lost their structure and acquired a round shape that impaired the orderly mosaic in some regions (Fig. 2). In Wistar rats, the destructive alterations of RPE cells (hypertrophy, hyperplasia, changes of the cell shape, or accumulation of lipofuscin granules) were observed at the age of 18 months, but they were significantly less pronounced as compared to OXYS rats (Fig. 2).

Near the hypertrophic RPE cells of OXYS rats, we often detected regions with a large number of small cells (Fig. 2B,C). We interpret this proliferation of small cells at the centre of the retina as fibrosis replacing lost RPE cells. Such localised scarring is typical at late stages of AMD. Moreover, the increase in RPE autofluorescence resulting from accumulation of lipofuscin granules was observed in OXYS rats at the age of 18 months (Fig. 2B,C and D).

### The ratio of mono- to binucleate RPE cells decreased in OXYS rats

Normally, in rodents (in contrast to humans), mono- and binucleate cells are present in the RPE; this phenomenon is attributed to the functional differentiation^25^ and to multinucleate cells, whose appearance is believed to be a sign of pathological changes of the retina^26^.

We found that at the age of 20 days, the percentages of mono- and binucleate cells in the RPE in OXYS rats differed from that in Wistar rats: the percentage of mononucleate cells significantly decreased and the percentage of binucleate cells increased in OXYS rats (p < 0.01; Fig. 1D,E); accordingly, the ratio of these cell types (mono/binucleate) decreased. Already at the age of 20 days in OXYS rats, there were multinucleate RPE cells (0.16% of the total number; Fig. 1F). The ratio of mononucleate to binucleate RPE cells was lower in OXYS rats than in Wistar rats at ages 3 and 18 months and did not change during the progression of retinopathy (Fig. 1C).

The percentage of multinucleate cells was almost twofold higher at the age of 3 months than in 20-day-old animals. At this age, the multinucleate cells were detected in the RPE of Wistar rats, but their proportion was significantly less than that in OXYS rats (Fig. 1F). At the age of 18 months in Wistar rats, the proportion of mononucleate cells was lower, but the percentages of binucleate and multinucleate cells were higher than those at 3 months of age (p < 0.05; Fig. 1D and E). The ratio of mononucleate to binucleate cells decreased, approximating the level in OXYS rats (Fig. 1C).

### Iba1^+^ retinal cells: morphology and distribution

**Figure 3:**
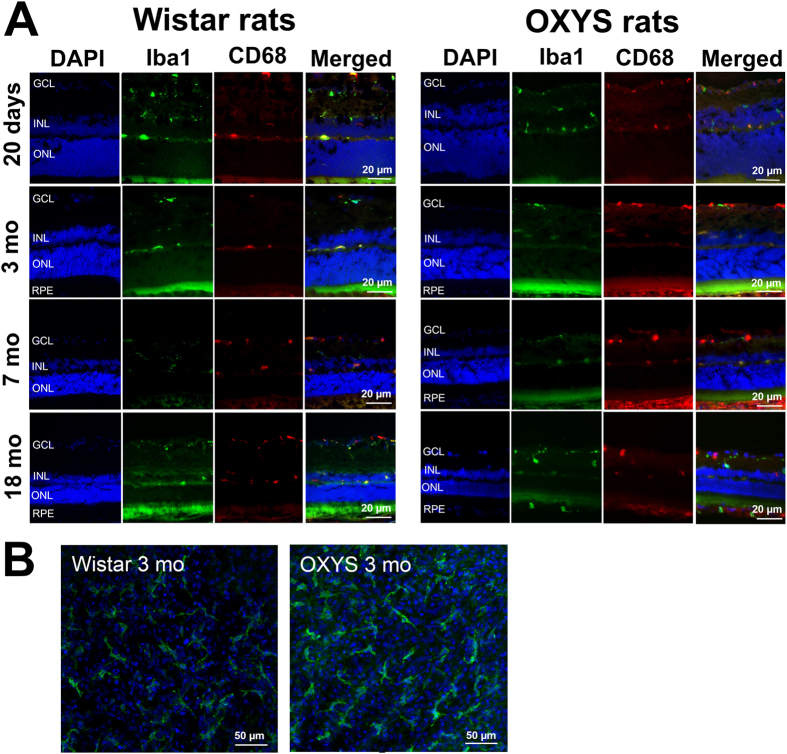
(**A**) Representative immunofluorescent images of co-localisation of microglia (Iba1, green), activated macrophages (CD68, red) and cell nuclei (DAPI, blue) in the retina of OXYS and Wistar rats at various ages. (**B**) Confocal images of a flat mount of the retinas (from 3-month-old OXYS and Wistar rats) showing the ramified Iba1^+^ microglial morphology.

**Figure 4:**
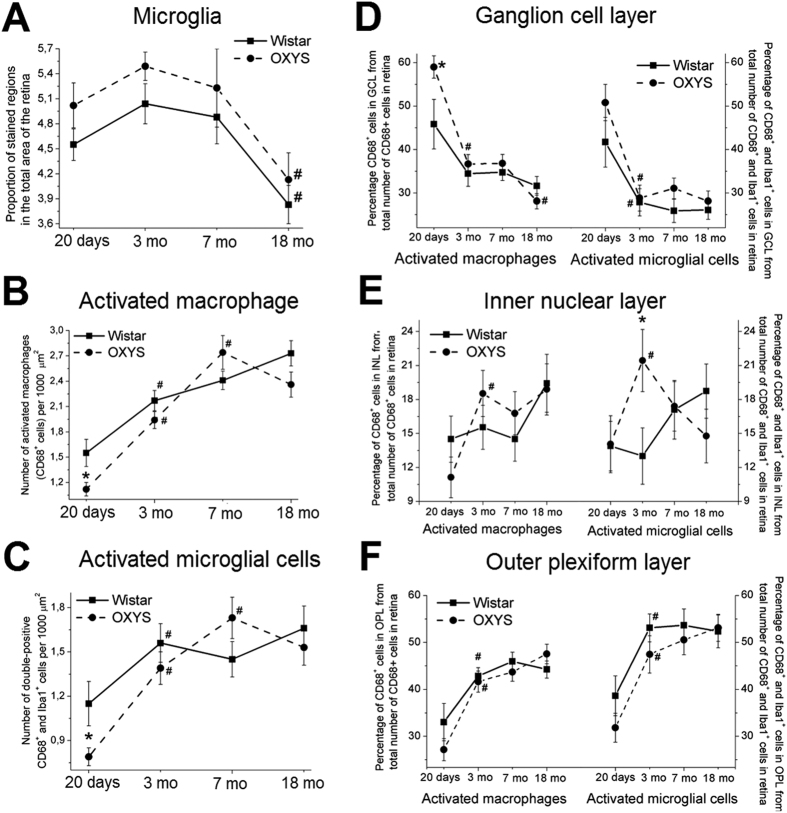
Analysis of alterations of Iba1^+^ microglia and CD68^+^ macrophage density as a function of age in the retina of OXYS and Wistar rats. (**A**) The data on immunofluorescence levels of the microglia marker Iba1 in retinal cryosections of 20-day-old and 3-, 7- and 18-month-old Wistar and OXYS rats. We calculated the proportion of stained regions in the total area of the retina. (**B**) The change in the number of activated macrophages (CD68^+^ cells) per 1000 μm^2^ in OXYS and Wistar retinas at different ages. (**C**) The change in the number of activated microglial cells (CD68^+^ and Iba1^+^ cells) per 1000 μm^2^ in OXYS and Wistar rats at various ages. The distribution of activated macrophages and microglial cells in various layers of the retina: ganglion layer (**D**), inner nuclear layer (**E**) and outer plexiform layer (**F**). The data are shown as mean ± SEM; *p < 0.05 for differences between the strains; ^#^p < 0.05 for age-related differences within a strain. Abbreviations: GCL, ganglion layer; INL, inner nuclear layer; ONL, outer plexiform layer; RPE, retinal pigment epithelium.

Next, we immunostained retinal samples from Wistar and OXYS rats at the ages of 20 days and 3, 7 and 18 months with antibodies against the Iba1 protein (a marker of microglia) to assess the state of microglia (Fig. 3A). The microglial cells were located in the ganglion layer, inner nuclear layer, inner plexiform layer, and outer plexiform layer. We detected no migration of microglia into the photoreceptor layer (characteristic of AMD) in OXYS rats. In addition, most microglial cells in Wistar and OXYS rats of all ages had ramified morphology (Fig. 3B). Quantitative analysis revealed a significant decrease in the proportion (%) of the Iba1^+^ area in the retina of OXYS and Wistar rats by the age of 18 months (p < 0.05), without significant differences between the strains (Fig. 4A).

### CD68^+^ retinal cells: distribution in the layers of retina

For analysis of the expression of CD68, which is expressed on the lysosomal membrane of active phagocytic cells in retinal microglia, we immunostained retina samples from Wistar and OXYS rats at ages 20 days and 3, 7 and 18 months (Fig. 3A).

Just as microglial cells, CD68^+^ cells were detected in the ganglion layer, inner nuclear layer, inner plexiform layer, and outer plexiform layer, but the migration of macrophages into the photoreceptor layer of the retina was not noted in any of the groups of animals under study. According to quantitative analysis, at the age of 20 days, the number of CD68^+^ retinal cells in OXYS rats was less than that in Wistar rats (p < 0.05; Fig. 4B). In Wistar rats, the number of CD68^+^ retinal cells gradually increased by age 3 months (p < 0.01) and at age 18 months was higher than that at 3 months of age (p < 0.01; Fig. 4B). The number of CD68^+^ retinal cells in OXYS rats gradually increased with age, from 20 days to 7 months, but we detected no significant differences between 7- and 18-month-old OXYS rats: when retinopathy actively progresses (Fig. 4B). Moreover, we observed a tendency for a decrease in the number of CD68^+^ cells in the retina of 18-month-old OXYS rats in comparison with age-matched controls (p = 0.07; Fig. 4B).

At the age of 20 days, the proportion (%) of CD68^+^ cells in the ganglion layer (relative to the total retina) in OXYS rats was significantly higher than that in Wistar rats (p < 0.01; Fig. 4D) and decreased with age and with progression of retinopathy, but the proportion of CD68^+^ cells in the outer plexiform layer increased (Fig. 4F; Table 1). The proportion of all retinal CD68^+^ cells in the inner nuclear layer of OXYS rats increased by the age of 3 months, without further changes (Fig. 4E). At the age of 18 months, we saw a tendency for a decrease in the number of CD68^+^ cells in the ganglion layer in OXYS rats compared to Wistar rats (p = 0.07; Fig. 4D).

### CD68^+^ and Iba1^+^ retinal cells: co-localisation and distribution

For analysis of the functional state of microglial cells, we used double staining for CD68 and Iba1 in the retina of rats at different ages (Fig. 3). We found that at the age of 20 days, the number of activated microglial cells in the retina of OXYS rats was 43.21% lower than that in Wistar rats (p < 0.05). At the age of 3 months in Wistar and OXYS rats, the number of activated microglial cells gradually increased to approximately the same level; this number remained unchanged in Wistar rats at ages 7 and 18 months. In OXYS rats during the development of retinopathy, there was a further increase in the number of activated microglial cells at the age of 7 months, but we observed no significant difference (Fig. 4C). The percentage of double-positive Iba1^+^ CD68^+^ cells relative to the total number of double-positive Iba1^+^ cells and CD68^+^ cells was sufficiently high – in the range of 61% to 73% in all the groups of rats (data not shown) – and did not change with age in OXYS and Wistar rats, indicating that the majority of activated macrophages in the retina were microglial cells.

The distribution of the activated microglial cells in the layers of retina was similar to the distribution of CD68^+^ cells in both animal strains: at the age of 20 days, the activated microglial cells were located predominantly in the ganglion layer; in this case, the percentage of activated microglial cells was significantly higher in OXYS rats than in Wistar rats (p < 0.05). By the age of 3 months in Wistar and OXYS rats, the percentage of activated microglial cells in the ganglion layer significantly decreased as compared to 20-day-old rats, and reached the level of approximately 27–28% (p < 0.01); there was no change with age (Fig. 4D). From age 20 days to 3 months in Wistar and OXYS rats, the percentage of activated microglial cells in the outer plexiform layer increased (p < 0.01; Fig. 4F). No alterations were observed in the inner nuclear layer in Wistar rats. In OXYS rats, the sudden increase in the percentage of activated microglial cells took place during manifestation of the first signs of retinopathy – at the age of 3 months – and we detected significant differences between the strains of rats (p < 0.05) and between ages (p < 0.05; Fig. 4E; Table 2).

### Increased expression of GFAP in Müller cells of OXYS rat retinas

**Figure 5:**
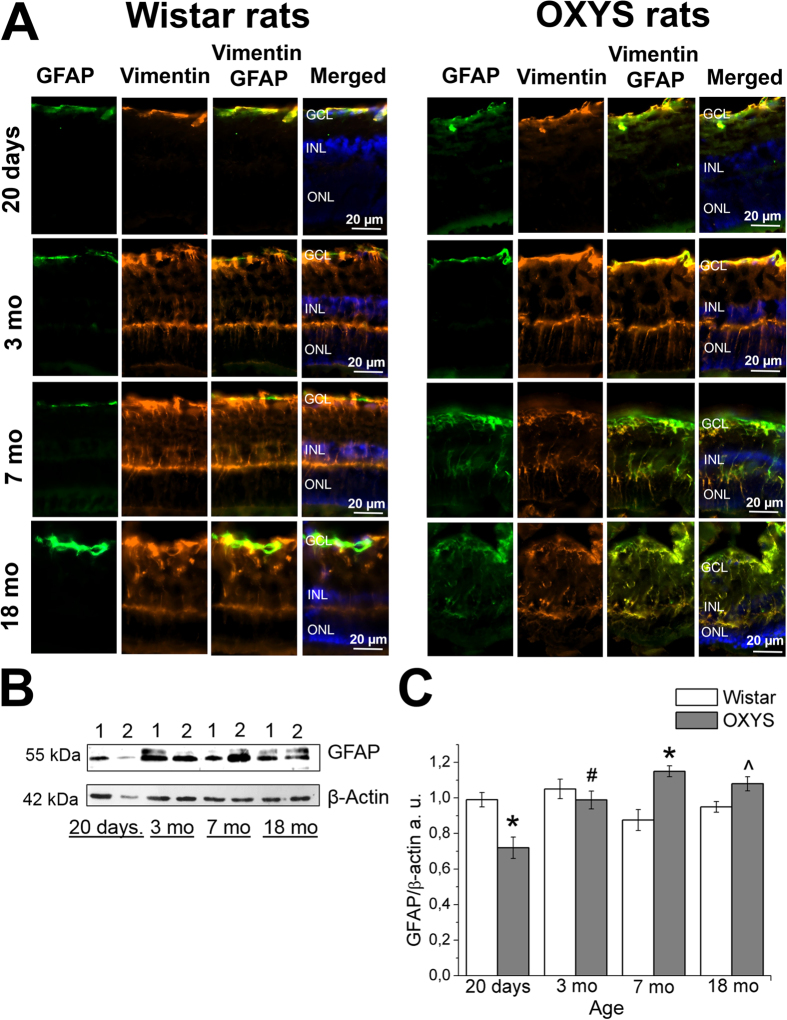
Age-related alterations of vimentin and GFAP expression in the rat retina. (**А**) Representative immunofluorescent images of co-localisation of GFAP (green), vimentin (yellow) and cell nuclei (DAPI, blue) in the retina of OXYS and Wistar rats at ages 20 days and 3, 7 and 18 months. Abbreviations: GCL, ganglion layer; INL, inner nuclear layer; ONL, outer nuclear layer. (**B**) Levels of GFAP protein in the retina of Wistar rats (lane 1) and OXYS rats (lane 2; 20-day-old and 3-, 7- and 18-month-old rats) according to immunoblot analysis. The relative amount of the GFAP protein was calculated as intensity of a GFAP band divided by intensity of a β-actin band in the retina (**C**). The data are shown as mean ± SEM; *p < 0.05 for differences between the strains; ^#^p < 0.05 for age-related differences within a strain; ^an insignificant difference between the strains.

To estimate the effects of macroglia – astrocytes and Müller cells – on the development of AMD-like retinopathy, we performed analysis of the dynamics of age-related changes in the expression of glial fibrillary acidic protein (GFAP; an intermediate-filament protein) in OXYS and Wistar rats at different ages (20 days, 3, 7 and 18 months; Fig. 5A). GFAP expression in Müller cells is an indicator of tissue stress and has been found to be associated with retinal degeneration, whereas the intermediate-filament protein vimentin is ubiquitously expressed in Müller cells of many mammalian species^27^. Figure 5A shows the immunohistochemical localisation of GFAP and vimentin in vertical retinal sections and western blot analysis of the GFAP protein in OXYS and Wistar rats (Fig. 5B,C). The western blotting revealed significantly decreased GFAP protein levels in 20-day-old OXYS rats, but this level increased at age 7 months as compared to Wistar rats (p < 0.05). This age-related GFAP overexpression in OXYS rats was accompanied by greater GFAP immunoreactivity in Müller cells (Fig. 5A). In Müller cells of normal retinas, vimentin immunostaining is distributed throughout the entire processes, from their end feet up to the outer retina, whereas GFAP immunoreactivity is limited to the inner margin of the retina^27^. In the present study, the co-localisation of vimentin and GFAP was negligible at the ages of 20 days and 3 months in both OXYS and Wistar rats. Nevertheless, OXYS rats’ retinas showed intense GFAP immunoreactivity in Müller cells at 7 and 18 months of age (Fig. 5A). Double staining for vimentin and GFAP revealed extensive co-localisation of the two intermediate-filament proteins, and vimentin/GFAP-positive apical processes of Müller cells extended into the ONL (Fig. 5A). As for Wistar rats, we detected small partial activation of Müller cells only at age 18 months.

## Discussion

There is now convincing evidence that alterations of RPE cells and of glial reactivity are common and early hallmarks of retinal degenerative diseases including AMD. Here, we used OXYS rats as a model and demonstrated that the first aberrations in the RPE and glia appear at the age of 20 days when postnatal development of the rat retina finishes and there are no clinical signs of AMD-like retinopathy in OXYS rats.

During the first 2 weeks of postnatal development after the completion of cell production in the rodent RPE, some cells undergo incomplete cell division (without cytokinesis) resulting in formation of binucleate cells, predominantly in the central area of the RPE^25^. As a result, many rodent RPE cells are binucleate. The mechanisms and physiological meaning of this phenomenon are unclear. It was proposed that the formation of binucleate RPE cells is related to eye growth and/or specialisation of RPE cells^25^. Recently, Mei Chen *et al*. published the data showing that age-related oxidative stress may promote failure of cytokinesis and multinucleation in RPE cells^26^. We detected emergence of the multinucleate RPE cells after the age of 20 days. The ratio of mononucleate to binucleate RPE cells in OXYS rats stayed lower than in Wistar rats both during the manifestation and progression of the disease. Nonetheless, the percentage of multinucleate RPE cells increased with age and remained higher than that in Wistar rats. In Wistar rats, these parameters changed in the same direction but only at the age of 18 months did they reach the level of 20-day-old OXYS rats; this situation allows us to regard these changes as typical signs of aging. It is possible that such changes reflect an impaired proliferation ability of RPE cells in OXYS rats already at the age of 20 days; this problem possibly promotes the development of destructive alterations in the retina at the age 3 months and subsequent progression with age. This interpretation is supported by data on the increase in the proportion of multinucleate RPE cells in several animal models^28^,^29^,^30^,^31^ and in the retina of patients with AMD^32^,^33^. It should also be noted that the manifestation of retinopathy in OXYS rats takes place simultaneously with the emergence of the (typical for AMD) atrophic and destructive changes in RPE cells: hyperplasia, hypertrophy and disruption of the orderly mosaic structure. These aberrations are increasing with age and with progression of retinopathy.

It is well known that AMD has features of chronic retinal inflammation^14^; alterations of the retinal glial phenotype with aging may promote both initiation and progression of the disease^11^. Macroglia in the human retina consist of Müller cells and astrocytes, which in addition to the trophic function promote contact between neighbouring neurons and participate in the formation of outer and inner limiting membranes^34^. At the age 20 days, the protein level of GFAP – a marker of Müller cells and astrocytes – was lower in the retina of OXYS rats than in age-matched Wistar rats. During completion of retinal development in the early ontogenesis, elimination of neurons takes place and there is selection of appropriate interneuronal connections by apoptosis. Previously, we demonstrated that apoptosis increases in the retina of OXYS rats at the age 20 days, but the expression of genes involved in the development process decreased (high-throughput RNA sequencing [RNA-Seq] data)^35^. We believe that the process of retinal maturation is disrupted in OXYS rats. The changes in macroglial cells that were identified in this study may lead to the disruption of trophic function and promote apoptosis of cells in the retina of 20-day-old OXYS rats.

Many diseases of retina are related to the gliosis of Müller cells and astrocytes^6^, and it is reported that the increased expression of intermediate filaments (including GFAP) is associated with formation of drusen in AMD^8^. It should be noted that upregulation of GFAP is a well-established indicator of retinal injury and reactive gliosis^36^. We did not detect macroglia activation in the period of manifestation of clinical signs of retinopathy in OXYS rats (age 3 months). Nevertheless, during active progression of the disease – ages 7 and 18 months, when most rats show alterations similar to third-stage AMD in humans, GFAP expression was increased in the retina.

It is known that microglial cells are activated at the basal level during healthy aging and express higher levels of major histocompatibility complex II (MHC II), CD11b and proinflammatory cytokines (such IL-1β, TNF-α and IL-6)^11^,^12^. The activation of microglial cells, their acquisition of an amoeboid/phagocytic morphology and migration into the photoreceptor layer of the retina (as migration of activated macrophages) contribute to a chronic parainflammatory state that is defined as a condition of a tissue adaptive response and is implicated in both initiation and progression AMD and other neurodegenerative diseases^14^,^37^,^38^. Enhanced accumulation of microglia in the subretinal space is accompanied by degenerative changes described in many mouse models of AMD^39^,^40^,^41^,^42^,^43^. Paradoxically, we did not observe the migration of activated macrophages and microglia into the photoreceptor layer either in the period of manifestation or progression of AMD-like retinopathy in OXYS rats. By contrast, at the preclinical stage – age 20 days – the number of activated macrophages was greater in the OXYS retina than in the retina of Wistar rats. With age, the migration of activated macrophages and microglial cells into the inner layers of neuroretina increased in both strains of rats, but the increase in the number of activated microglial cells in the inner nuclear layer was found only in OXYS rats at age 3 months: the period of active manifestation of clinical signs of AMD-like retinopathy. It is noteworthy that previously, only at this age, in OXYS rats’ retina, we have identified the increased protein expression of inducible nitric oxide synthase (iNOS), which produces large quantities of NO as part of an immune defence mechanism^44^. The inner nuclear layer contains bipolar cells, horizontal cells, amacrine cells and Müller cells. Perhaps the increased migration of microglia into the inner nuclear layer is due to the fact that the amacrine and bipolar neurons are the most sensitive cells among the associative neurons in the OXYS rat retina and are the first to undergo degenerative changes during the development of AMD-like retinopathy in OXYS rats^16^.

Overall, these and our previously obtained data suggest that AMD-like pathology in OXYS rats in general may derive from an imbalance of immune processes, including alterations in inflammation. Previously, pathway analysis of RNA-Seq data from the retina of OXYS and Wistar rats (at ages 20 days and 3 and 18 months)^20^,^35^ revealed significant downregulation of immune-system genes in OXYS rat retinas; this list includes many regulators of immunity, such as leukocyte markers, chemokines, cytokines, complement components, interferon-inducible proteins, and MHC (major histocompatibility complex) genes. The possible causes of the immune imbalance are the accelerated thymus involution and a decline of T-cell-mediated immunity in OXYS rats. Progressive involution of the thymus, a hallmark of aging, leads to deterioration of those immune functions that are related to T-cell-dependent immunity. Because immunosenescence is defined as progressive and generalised deterioration of immune functions that affects all cells and organs of innate and adaptive immunity, it can be assumed that the immune imbalance (most likely provoked by accelerated thymic involution) can create a specific metabolic background for the development and progression of AMD-like retinopathy as well as many other manifestations of accelerated senescence in OXYS rats. Nevertheless, the results of our present study on the contribution of age-related alterations in the glia to the development of AMD-like pathology in OXYS rats (unlike data on the RPE) are not quite consistent with pathological changes typical of people with AMD. These results, as well as the wide spectrum of age-related retinal alterations in humans and differences in the risk of AMD may be attributed to genetic differences and differences in the underlying molecular events.

## Materials and methods

### Animals

Male senescence-accelerated OXYS rats (n = 42) and age-matched male Wistar rats (n = 42) at ages 20 days and 3, 7 and 18 months were obtained from the Breeding Experimental Animal Laboratory of the Institute of Cytology and Genetics, the Siberian Branch of the Russian Academy of Sciences (Novosibirsk, Russia). For immunohistochemical analysis, at least six rats were used from each age group. At least four tissue slices were analysed per animal. For western blot analysis, four rats were used from each age group. The OXYS rat strain was derived from the Wistar rat strain at the Institute of Cytology and Genetics as described earlier^45^ and was registered in the Rat Genome Database (http://rgd.mcw.edu/). At this point, we have the 109th generation of OXYS rats with spontaneously developing accelerated senescence syndrome including AMD-like retinopathy inherited in a linked manner.

All animal procedures and experimental protocols were approved by Institutional Review Board of the Institute of Cytology and Genetics, according to The Guidelines for Manipulations with Experimental Animals. At the age of 4 weeks, the pups were weaned and housed in groups of five animals per cage (57 × 36 × 20 cm) and kept under standard laboratory conditions (22 °C ± 2 °C, 60% relative humidity, and natural light). The rats were provided with standard rodent feed, PK-120-1, Ltd. (Laboratorsnab, Russia) and given water *ad libitum*.

### RPE flat-mount staining

OXYS rats and Wistar rats were euthanised by CO_2_ asphyxiation; the eyes were carefully excised and fixed in 4% paraformaldehyde in phosphate-buffered saline (PBS) for 2 h. The anterior segment of the eye (cornea, iris, ciliary body and lens) was removed. Retinal tissue was carefully excised from the eyecup, and the remaining cups containing RPE, choroid, and sclera were thoroughly washed and processed for immunofluorescent staining. The RPE, choroid and sclera tissues were permeabilised with 1% Triton X-100 at room temperature for 10 min and incubated with fluorescein isothiocyanate (FITC)-phalloidin (1:500, P5282, Sigma-Aldrich, USA) at 4 °C overnight to visualise the cytoskeleton and cell shapes during en face imaging. After thorough washes, the slices were flat-mounted on glass slides and were coverslipped with the Fluoro-shield mounting medium containing 4′,6-diamidino-2-phenylindole (DAPI; ab104139, Abcam, Cambridge, UK). All the samples were examined under the confocal laser microscope LSM 510 META (Zeiss, Germany).

### Antibodies

Rabbit polyclonal anti-GFAP (ab7260), rabbit polyclonal anti-actin (ab1801), goat polyclonal anti-Iba1 (ab5076), mouse monoclonal anti-CD68 (ab31630), chicken polyclonal anti-vimentin (ab24525) and a secondary antibody – a donkey anti-goat IgG H&L antibody (conjugated with Alexa Fluor^®^ 488; ab150129), donkey anti-rabbit IgG H&L antibody (conjugated with Alexa Fluor^®^ 488; ab150073), donkey anti-mouse IgG H&L antibody (conjugated with Alexa Fluor^®^ 568; ab175472) and goat anti-rabbit IgG H&L antibody (HRP; ab6721) were acquired from Abcam (Cambridge, UK).

### Western blotting

Immunoblotting was performed as previously described^22^. Antibodies and dilutions used in this study included an anti-GFAP antibody (1:1,000) and anti-β-actin antibody (1:1,000). After blockage with 5% bovine serum albumin (BSA; Sigma-Aldrich) in 0.01 M phosphate buffer with 0.1% Tween-20 (PBS-T) for 1 h, the membranes were incubated with the primary antibodies at 4 °C overnight. After incubation with the respective secondary antibody (1:3,000), chemiluminescent signals were measured and scanned, and intensity of the bands was quantified in the ImageJ software (NIH, Bethesda, MD, USA). β-Actin always served as an internal loading control.

### Immunofluorescent staining of frozen slices

Immunofluorescent staining was performed according to the standard indirect method as described elsewhere^19^. Primary antibodies and dilutions were as follows: anti-Iba1 (1:250), anti-GFAP (1:250), anti-vimentin (1:250) and anti-CD68 (1:300). Primary antibodies were incubated for 18 h at 4 °C. After incubation with the respective secondary antibodies diluted 1:300 for 1 h at room temperature, the tissue slices were coverslipped with the Fluoro-shield mounting medium containing DAPI (ab104139, Abcam, Cambridge, UK) and were examined under the microscope Axioplan 2 (Zeiss, Germany). Quantitative densitometric analyses were performed on digitised images in the ImageJ software.

### Statistical analysis

The data were subjected to analysis of variance (ANOVA in the Statistica 8.0 software). The *post hoc* test was applied to significant main effects and interactions in order to assess the differences between some sets of means. The data are shown as mean ± SEM. The differences were considered statistically significant at p < 0.05.

## Additional Information

**How to cite this article:** Telegina, D. V. *et al*. Contributions of age-related alterations of the retinal pigment epithelium and of glia to the AMD-like pathology in OXYS rats. *Sci. Rep.* **7**, 41533; doi: 10.1038/srep41533 (2017).

**Publisher's note:** Springer Nature remains neutral with regard to jurisdictional claims in published maps and institutional affiliations.

## Figures and Tables

**Table 1:** The percentage ratio of activated macrophages (CD68^+^ cells) in various layers of the retina to the total number of CD68^+^ cells in the retina of OXYS and Wistar rats.

Strain	Age	GCL	IPL	INL	OPL	ONL
Wistar	20 days	41.66 ± 4.2	5.15 ± 1.36	14.5 ± 2.04	33.02 ± 3.07	0
Wistar	3 mo	34.45 ± 2.92	6.73 ± 1.13	15.56 ± 1.95	**42.84 ± 1.79**#	0.64 ± 0.38
Wistar	7 mo	34.74 ± 1.9	**3.37 ± 0.82**#	14.51 ± 1.98	45.94 ± 2.00	1.19 ± 0.50
Wistar	18 mo	31.64 ± 2.16**	4.05 ± 1.07	19.43 ± 2.56	44.22 ± 1.22**	0.67 ± 0.47
OXYS	20 days	58.98 ± 2.55*	1.73 ± 0.74*	11.12 ± 1.79	27.15 ± 2.35	0.55 ± 0.38
OXYS	3 mo	**36.65 ± 2.16**#	2.86 ± 0.7*	**18.52 ± 2.04**#	**41.63 ± 2.25**#	0
OXYS	7 mo	36.08 ± 2.09	0.67 ± 0.39*	16.79 ± 1.91	43.69 ± 1.98	1.67 ± 0.52
OXYS	18 mo	**28.12 ± 1.76**#,+,**	**4.13 ± 1.02**#	18.89 ± 2.26	47.55 ± 2.10**	1.03 ± 0.49

The data are shown as mean ± SEM; ^*^p < 0.05 for differences between the strains, ^#^p < 0.05 for age-related differences within a strain, ^p < 0.05 for age-related differences compared to rats at the age of 3 months, ^**^p < 0.05 for age-related differences compared to 20-day-old rats, ^+^an insignificant tendency between the strains. Abbreviations: GCL, ganglion layer; IPL, inner plexiform layer; INL, inner nuclear layer; OPL, outer plexiform layer; ONL, outer nuclear layer.

**Table 2:** The percentage ratio of activated microglia (double-positive CD68^+^ and Iba1^+^ cells) in various layers of the retina to the total number of double-positive CD68^+^ and Iba1^+^ cells in the retina of OXYS and Wistar rats.

Strain	Age	GCL	IPL	INL	OPL	ONL
Wistar	20 days	37.21 ± 3.82	4.85 ± 1.41	13.89 ± 2.19	38.61 ± 4.64	0.95 ± 0.95
Wistar	3 mo	**27.85 ± 3.11**#	5.81 ± 1.40	13.00 ± 2.48	**53.08 ± 2.7**#	0.26 ± 0.26
Wistar	7 mo	25.89 ± 2.69	2.75 ± 1.98	17.09 ± 2.59	53.65 ± 3.45	0.64 ± 0.50
Wistar	18 mo	26.08 ± 2.14	3.43 ± 1.29	18.73 ± 2.39	52.34 ± 3.50	0
OXYS	20 days	50.78 ± 4.18*	2.20 ± 1.10	14.05 ± 2.51	31.83 ± 31.07	0.76 ± 0.52
OXYS	3 mo	**28.83 ± 2.98**#	2.98 ± 1.17*	21.44 ± 2.74*,#	**47.45 ± 3.99**#	0
OXYS	7 mo	31.05 ± 2.40	0.22 ± 0.22*	17.39 ± 2.19	50.52 ± 3.18	0.81 ± 0.57
OXYS	18 mo	**28.13 ± 2.35**#	2.27 ± 0.82	**14.77 ± 2.37**^	53.11 ± 2.87	0.86 ± 0.86

The data are shown as mean ± SEM; ^*^p < 0.05 for differences between the strains, ^#^p < 0.05 for age-related differences within a strain, ^p < 0.05 for age-related differences compared to rats at the age of 3 months. Abbreviations: GCL, ganglion layer; IPL, inner plexiform layer; INL, inner nuclear layer; OPL, outer plexiform layer; ONL, outer nuclear layer.
